# Disparities in ACL Reconstruction: the Influence of Gender and Race on Incidence, Treatment, and Outcomes

**DOI:** 10.1007/s12178-021-09736-1

**Published:** 2021-12-31

**Authors:** Sai K. Devana, Carlos Solorzano, Benedict Nwachukwu, Kristofer J. Jones

**Affiliations:** 1 Department of Orthopaedic Surgery, University of California, Los Angeles, USA; 2 Department of Orthopaedic Surgery, Hospital for Special Surgery, New York City, USA

**Keywords:** ACL disparities, ACL gender differences

## Abstract

**Purpose of Review:**

Anterior cruciate ligament (ACL) rupture is a common injury that has important clinical and economic implications. We aimed to review the literature to identify gender, racial and ethnic disparities in incidence, treatment, and outcomes of ACL injury.

**Recent Findings:**

Females are at increased risk for ACL injury compared to males. Intrinsic differences such as increased quadriceps angle and increased posterior tibial slope may be contributing factors. Despite lower rates of injury, males undergo ACL reconstruction (ACLR) more frequently. There is conflicting evidence regarding gender differences in graft failure and ACL revision rates, but males demonstrate higher return to sport (RTS) rates. Females report worse functional outcome scores and have worse biomechanical metrics following ACLR. Direct evidence of racial and ethnic disparities is limited, but present. White athletes have greater risk of ACL injury compared to Black athletes. Non-White and Spanish-speaking patients are less likely to undergo ACLR after ACL tear. Black and Hispanic youth have greater surgical delay to ACLR, increased risk for loss to clinical follow-up, and less physical therapy sessions, thereby leading to greater deficits in knee extensor strength during rehabilitation. Hispanic and Black patients also have greater risk for hospital admission after ACLR, though this disparity is improving.

**Summary:**

Females have higher rates of ACL injury with inconclusive evidence on anatomic predisposition and ACL failure rate differences between genders. Recent literature has suggested inferior RTS and functional outcomes following ACLR in females. Though there is limited and mixed data on incidence and outcome differences between races and ethnic groups, recent studies suggest there may be disparities in those who undergo ACLR and time to treatment.

## Introduction

Differences in race, ethnicity, gender, and socioeconomic status within the diverse population of the USA have led to the permeation of disparities across many different specialties in medicine and surgery [1–3]. Within orthopedic surgery in particular, disparities in utilization, selection, treatment, and outcomes have been shown in multiple subspecialties [4–9]. Here, we review the existing evidence in the literature pertaining to disparities in anterior cruciate ligament (ACL) injury, management, and outcomes. ACL injury is the most common isolated ligamentous injury that occurs in athletes, with over 100,000 ACL reconstructions performed per year and over three billion dollars in estimated costs annually in the USA alone [10–13]. The profound clinical and economic impact of ACL injuries highlights the importance of understanding and addressing disparities that exist to ensure adequate patient care.

## Gender Disparity in ACL Injury, Treatment, and Outcomes

Compared to male counterparts within the same sport, it has been well established that female athletes are two to eight times more likely to sustain an ACL injury [14–16]. With the increase in participation in youth athletics as well as expansion of women’s collegiate and professional programs over the last few decades [17], it has become increasingly important to better understand potential mechanisms underlying the gender difference in injury rates.

### Intrinsic Risk Factors and Predisposition to Injury

Anatomic variation in the femoral intercondylar notch (ICN) has been studied as a possible intrinsic risk factor contributing to the discrepancy in ACL injury between male and female athletes. Notch width index (NWI) is a measure that attempts to standardize notch width relative to overall distal femoral width and is commonly used to define the size of the ICN. Compared to males, females have smaller NWI and smaller standardized ACL cross-sectional area [18, 19]. While ICN stenosis has been shown to be associated with an increased risk of noncontact ACL injury [20], there is conflicting data surrounding the significance of NWI differences between males and females in the context of ACL injury [21–23]. A study of 108 ACL injury radiographs compared to controls showed a higher proportion of A-shaped notches (notch narrows from base to midsection and apex) in females relative to males; however, notch shape and sex did not correlate with injury status [24]. Similarly, there have been conflicting findings regarding the association of notch size and ACL size [22, 25]. In summary, there appears to be a difference in ICN size and ACL size between genders without conclusive correlation to injury (Table 1).

**Table 1:** Summary of knee anatomic differences between males and females

Quadriceps angle	Up to 5.8° greater in females compared to males leading to a more laterally directed pull of the quadriceps, which may place the ACL at higher risk for injury [18, 26]
Intercondylar notch	Although female patients have smaller notch width indices than males, there is conflicting data surrounding the significance of differences in notch width in the context of ACL injuries [18–23].
ACL size	When standardized for body weight and height, females have a smaller ACL size and cross-sectional area than males; however, there is no conclusive correlation to ACL injury [19, 22, 25].
Tibial slope	There is currently mixed data surrounding the significance of posterior tibial slope on differences in ACL injury rates between males and females [27–30].

Recently, much emphasis has been placed on the geometry of the knee, specifically the quadriceps angle (Q angle) and posterior tibial slope (PTS). The Q angle has been shown to be up to 5.8° greater in females compared to that in males leading to a more laterally directed pull of the quadriceps, which may place the ACL at higher risk for injury [18, 26]. It is widely accepted that an increase in PTS places the tibia more anterior relative to the femur during quadriceps contraction, which may result in increased strain on the ACL. This has been supported by multiple studies that have demonstrated increased risk of ACL injuries in those with higher PTS.

Hohmann et al. found that PTS of the medial plateau was higher in ACL-injured females than males [27]. Similarly, Terauchi et al. found that medial plateau PTS was significantly greater in the ACL-deficient females compared to controls, whereas no significant difference was seen between the injured and control male groups [28]. In contrast, multiple studies have noted no significant difference in PTS measurements between men and women with ACL injury [29, 30]. Given the mixed results when comparing gender, it may be that the risk for ACL injury in relation to PTS is associated with a particular threshold measure rather than sex.

Estrogen, progesterone, and androgen receptors have been localized to fibroblasts and endothelial cells within the ACL, thereby suggesting a hormonal influence on susceptibility to ACL injury [31, 32]. A meta-analysis found that anterior knee laxity was greater in the ovulatory phase than in the luteal phase and lowest in the follicular phase [33]. Interestingly, ACL injury is more likely to occur in the early and late follicular phases [34, 35]. There is a lack of large-scale studies that compare hormone levels, markers of laxity, and injury between genders. Thus, the current data is insufficient to make any conclusive statement regarding the influence of hormonal and menstrual cycles on the rate of ACL injury in females.

Biomechanical and kinematic factors have also been extensively studied as possible explanations to gender differences in ACL injury. Females have a higher quadriceps-to-hamstring mass ratio and a higher ratio of quadriceps-to-hamstring recruitment [36]. Furthermore, females have been noted to land in a more erect posture with an external rotation position [19]. These factors have been theorized to increase the stress on the ACL. Females have also been shown to have higher recruitment of lateral thigh musculature and to consequently have up to a 2.5 times greater knee abduction moment [37]. While this has not been directly linked to ACL injury, these abduction moment differences arise after pubertal growth spurts around the time which the disproportionate increase in female injury rates occurs [38].

Ultimately, many physiologic, biomechanical, and kinematic differences have been established that may explain differences in ACL injury rates between males and females, which as it stands appears to be a multifactorial phenomenon.

### Treatment

Over the past few decades, there has been a significant increase in the rates of ACLR among both men and women in the USA and internationally [39–41]. A 2017 study showed that females aged 13–17 had the highest rate of ACLR, more than any other sex-age strata [42]. Despite higher rates of primary injury in female athletes, males have been reported to undergo ACLR more frequently [43, 44]. However, there has been an increase in the proportion of females undergoing ACLR compared to males in both ambulatory and inpatient settings [45]. Additionally, graft choice has previously been thought to be influenced by gender, with females more likely to receive an allograft as opposed to autograft revision ACLR [46]. However, Svantesson et al. subsequently reported no gender difference with graft choice in primary ACLR [47].

### Outcomes

Currently, there exists conflicting evidence regarding differences in graft failure between genders. While females have been shown to have greater laxity on post-operative physical exam [48], a multitude of studies have shown no significant difference in graft rupture/failure rates [49–54]. On the contrary, other studies have identified male sex as a risk factor for ipsilateral revision and graft failure following primary ACLR [55, 56]. Risk for contralateral ACLR also has conflicting evidence, with some studies identifying female sex as a risk factor [57–59], and others showing no significant difference between male and female patients [60, 61].

Another primary outcome of interest specifically in athletes that undergo ACLR is the overall time to return to sport (RTS). Young, male athletes have significantly higher RTS rates compared to female athletes at 12 months post primary ACLR (differences summarized in Table 2) [62, 63^•^]. One potential explanation is that male patients have been shown to have greater psychological readiness to RTS throughout the entire rehabilitation process compared to females [64, 65, 66^•^] and self-reported psychological readiness has been identified as the most significant factor in predicting subsequent return to comparable athletic performance [67]. This implies a potential role for psychological treatment as part of the post-ACLR rehabilitation programs and a need to further understand RTS protocols for athletes.

**Table 2:** Reported differences in return to sport between males and females

Study	Study variables	Methods	Findings
Hamrin Senorski [62]	RTS rates	Males and females; evaluated at 12 months after surgery for RTS via Tegner Activity Scale	Patients of male sex found to have favorable odds ratio for RTS after ACLR compared to females (OR, 2.58; 95% CI, 1.43–4.65; *P* = .0016)
Webster [63^•^]	RTS rates	Males and females; evaluated at 12 months after surgery for return to sport	Male athletes who were in the ≤ 25-year and 26- to 35-year age brackets had significantly higher return rates than female athletes (52% vs 39% and 37% vs 18%, respectively; *P* < .001), whereas no sex-based differences in RTS were seen after 36 years of age.
Kostyun [64]	Readiness to RTS	Males and females; assessed with ACL-RSI scores at pre-op, 3 months after surgery, and at RTS clearance	Female athletes reported lower readiness to RTS at all three time points of the study (*P* < 0.010).
Webster [66^•^]	Readiness to RTS	Males and females; participants evaluated after clearance for RTS, psychological readiness assessed with ACL-RSI scores	Male patients who participated frequently in sports before ACL injury had higher psychological readiness than females (*β* = 5.8; 95% CI, 2–10).
Webster [67]	Return to pre-injury performance, RTS	Males and females; patients followed out to a mean of 3 years to determine if they returned to pre-injury level of performance	Males and females had similar rates of return to pre-injury performance, as well as return to competition at 12 months after surgery.

In the current literature, male patients have superior functional outcomes following primary ACLR. Data from the Swedish knee ligament registry shows favorable outcomes for males for the Knee Injury and Osteoarthritis Outcome (KOOS) score and EuroQol EQ-5D health status measure [68]. Similar findings have been documented with self-reported knee questionnaires [69, 70]. Furthermore, male patients who return to work and drive sooner have greater odds of participating in moderate-to-vigorous physical activity after ACLR [71, 72].

In addition to RTS and functional outcomes, post-operative biomechanical testing also favors males. In both animal and human studies, females have decreased knee extensor muscle strength compared to males at 1 year after surgery, and slower rates of quadriceps torque development in the affected limb [73, 74^•^, 75–77]. Female athletes with ACLR also display worse Landing Error Scoring System (LESS) scores and are more likely than males to commit errors related to medial knee displacement during landing movement patterns [78]. The overall discrepancy in outcomes between genders, specifically for primary ACLR, highlights the importance of personalized rehabilitation protocols and further level I studies to elucidate the important modifiable risk factors both pre- and post-operatively.

## Disparities in Race and Ethnicity

### Predisposition to Injury

Anatomic variation in femoral ICN has been studied within patients of different races and ethnicities as a possible risk factor for ACL injury. Compared with African American males, Caucasian males consistently have narrower ICN width; however, there is conflicting evidence surrounding females of various races, with some studies showing significant differences between African American and White females, and others showing no difference [79, 80]. Additionally, no significant anatomic discrepancy has been identified in Q angle or PTS between White and African patients [81, 82]. Injury trends within the Women’s National Basketball Association (WNBA) help illustrate potential disparities by race, with White athletes having more than 6 times the ACL tear rate of other ethnic groups combined [83]. Data of Han Chinese patients with ACL injuries shows a higher proportion of patients with a small tibial footprint size compared to Western populations with ACL injuries, suggesting differences in tibial footprint size could play a role between races [84, 85]. There is a paucity of studies and limited data on racial and ethnic predisposition to ACL injury despite well-described anatomic differences.

### Treatment

Race and ethnicity have been shown to influence treatment following ACL injury*.* White patients are more likely than non-White patients to undergo ACLR after ACL injury diagnosis [86]. Language similarly influences rates of ACLR, as patients that speak English as a primary language are most likely to undergo surgical reconstruction, followed by multilanguage households, while exclusively Spanish-speaking patients are significantly less likely to undergo reconstruction [86]. Among pediatric and adolescent populations, Black and Hispanic patients experience greater surgical delays after ACL rupture [87^••^]. In the Kaiser Permanente ACL registry data, when controlling for insurance status, Asian, Hispanic, and Black patients were less likely to undergo elective ACL revision compared to White patients, despite having comparable access to care through Kaiser [88^••^]. Race distribution was significantly different across graft types used in the Kaiser data, with a lower proportion of White patients in the bone-tendon-bone (BTB) autograft group compared to the hamstring autograft or allograft groups [89]. Black high school and collegiate athletes are more likely to receive a BTB versus hamstring autograft [90]. In the post-operative period, Black and other non-White races have significantly increased risk of loss to clinical follow-up [91]. Additionally, young Black and Hispanic athletes also have less physical therapy sessions during the rehabilitation process, highlighting further racial disparity in ACL treatment [87^••^].

### Outcomes

There is limited and mixed evidence on the influence of race and ethnicity on post-operative complications following ACLR. Some studies have shown that White patients have a significantly higher risk than Hispanic, Asian, and Black patients for ipsilateral revision and contralateral ACLR [92–94] while others have found no difference in ipsilateral revision rates between races [87^••^, 95]. Hispanic ethnicity [96] and Black race [97] have been identified as risk factors for post-operative readmission following ACLR. However, between 2007 and 2015, readmission rates of Black and Hispanic patients have been decreasing relative to that of White patients [98^•^].

There is limited and thus inconclusive data for disparities in RTS and biomechanical testing. A study of 915 pediatric primary ACLRs showed no difference in RTS clearance by race, though Black and Hispanic patients were significantly less likely to be cleared to RTS overall [87^••^]. Another study did not find a significant difference between White and non-White patients in RTS rates at 2-year follow-up [99]. Isokinetic dynamometer testing has shown that Black and Hispanic pediatric and adolescent patients experience significantly greater quadriceps deficits at 6 and 9 months following ACLR, likely due in part to a discrepancy in physical therapy follow-up [87^••^]. Overall, while the studies are limited, racial and ethnic disparities exist in treatment and outcome and this is likely multifactorial, but implies the presence of biases that require further investigation.

## Where Do We Go from Here?

Gender differences are evident and call for gender-specific studies in order to better understand injury and pathophysiology and close the gap in outcomes between males and females. Given the much higher incidence of ACL injury in females, there is a demand for more studies focusing on injury prevention such as neuromuscular and proprioceptive training. Special attention is needed for young female athletes as the number of sports programs continues to increase. There is a paucity of literature on high-performance female athletes compared to available data for male athletes [100]. Limited understanding of differences in anatomy, physiology, and biomechanics along with applying male-dominant research to all is likely contributing to the inferior outcomes reported among females.

Health care providers across all specialties including orthopedic surgery show implicit biases related to race and gender, similar to those of the general public [101, 102]. Druckman et al. analyzed how sports medical staff responded to student-athlete case vignettes and showed that staff viewed Black athletes as having higher initial pain tolerance after ACL tear compared to their White peers [103]. While such biases have not been directly shown to effect outcomes, it is likely a contributory factor that has to be addressed at the individual level. Providers must make an effort to take into consideration not only race and gender, but also important social determinants of health, access to care, and patient expectations all of which can have a large impact on patient outcomes.

Lastly, it has been well documented that patient satisfaction and health outcomes are favorable when there is racial, ethnic, and linguistic concordance between the physician and patient [104–106]). Within orthopedic surgery residency programs, between 2006 and 2015, female representation increased from 10.9 to 14.4%, but this rate of increase was significantly lower than those of many other surgical specialties. Additionally, there was no significant improvement in racial/ethnic diversity among orthopedic residency programs over the same time period [107^•^]. A recent study similarly showed that Latino, African American, and Native American groups were significantly underrepresented in orthopedic residencies [108]. A more representative workforce from surgeon to physical therapists to athletic trainers may have a positive impact on disparities and treatment outcomes.

## Conclusion

There is broad evidence of disparities surrounding the incidence, treatment, and outcomes following ACL injury. Disproportionate rates of injury, anatomic differences, variable biomechanics, access to care, and implicit biases among other factors must be considered. While there is evidence of improvement in certain areas, we still have work to do to close the gap and optimize care for all.
